# The Insulin-Like Growth Factor System

**DOI:** 10.1155/EDR.2003.205

**Published:** 2003

**Authors:** Derek Le Roith

**Affiliations:** 1 Diabetes Branch Room 8D12 Building 10 National Institutes of Health MSC 1758 Bethesda MD 20892-1758 USA

## Abstract

The insulin-like growth factor (IGF) system in ubiquitous and plays a role in every tissue of the body. It is comprised of ligands, receptors and binding proteins, each with specific functions. While it plays an essential role in embryonic and post-natal development, the IGF system is also important in normal adult physiology. There are now numerous examples of diseases such as diabetes, cancer, and malnutrition in which the IGF system is a major player and, not surprisingly, there are attempts to affect these disorders by manipulating the system.


*Experimental Diab. Res.*, 4:205–212, 2003
Copyright © Taylor and Francis Inc.
ISSN: 1543-8600 print / 1543-8619 online
DOI: 10.1080/15438600390249664

### The Insulin-Like Growth Factor System

**Derek Le Roith**

*Diabetes Branch, National Institutes of Health, Bethesda, Maryland, USA*

**The insulin-like growth factor (IGF) system in ubiquitous and plays a role in every tissue of the body. It is comprised of ligands, receptors and binding proteins, each with specific functions. While it plays an essential role in embryonic and post-natal development, the IGF system is also important in normal adult physiology. There are now numerous examples of diseases such as diabetes, cancer, and malnutrition in which the IGF system is a major player and, not surprisingly, there are attempts to affect these disorders by manipulating the system.**

**Keywords** Binding Proteins; Insulin-Like Growth Factors; Receptors; Signaling Pathways

### INTRODUCTION

The insulin-like growth factor (IGF) system includes three ligands (insulin, IGF-I, and IGF-II), three receptors (the insulin receptor [IR], the IGF-I receptor [IGF-IR], and the mannose-6-phosphate IGF-II receptor [M6P/IGF-IIR]), as well as six IGF-binding proteins (IGFBPs). This family of growth factors has been extensively studied, because of its critical roles in both normal physiology and in various disease states, such as cancer, diabetes, and nutritional status abnormalities. The various components of the IGF family are widely expressed, and other important functions of this system are rapidly being discovered.

This article will present an overview of the IGF system, including the biological functions of its various components and how the expression levels of these components are controlled. The major emphasis will be on more recent findings, with particular focus on aberrations of the system, because many excellent reviews have previously covered the more familiar aspects of the IGF system (Baxter, 2000; Clemmons, 2001; Le Roith et al., 1995; Rajaram et al., 1997; Stewart and Rotwein, 1996). Where appropriate, aberrations of the system will be discussed in the context of diabetes and its complications.

### LIGANDS

### IGF-I

Insulin-like growth factor-I (IGF-I) is expressed by most tissues of the body. It circulates as a 70-residue single-chain polypeptide with four domains, designated as B, C, A, and D. In comparison, proinsulin includes the B, C, and A domains, whereas mature insulin produced and secreted by the pancreas includes only the B and A domains. Circulating IGF-I is primarily derived from the liver, although other tissues, such as fat, may also contribute (Yakar et al., 1999). The major factors that regulate hepatic IGF-I biosynthesis are growth hormone (GH), insulin, and nutritional status. Although GH is the major factor that stimulates IGF-I expression and release, insulin and nutrients can also significantly affect this response (Bichell et al., 1992; Kaytor et al., 2001; Zhang et al., 1998).

In extrahepatic tissues, IGF-I gene expression is regulated by several factors in addition to GH. For example, both prostaglandin $E_2$ ($PGE_2$) and parathyroid hormone (PTH) increase IGF-I mRNA levels in cultured osteoblasts, whereas GH has little effect on IGF-I expression in this system (Bichell et al., 1993). Estradiol also increases expression of IGF-I in osteoblasts (Ernst and Rodan, 1991). Other examples include angiotensin II regulation of IGF-I expression at the local level,

Received 20 December 2003; accepted 17 February 2003.
Address correspondence to Derek Le Roith, MD, PhD, Chief, Diabetes Branch, Room 8D12, Building 10, National Institutes of Health MSC 1758, Bethesda, MD 20892-1758, USA. E-mail: derek@helix.nih.gov by stimulating IGF-I production in the cardiovascular system (Brink et al., 1999), the inducion of IGF-I mRNA during compensatory renal growth (Mulroney et al., 1992), and in the regulation of skeletal muscle growth and repair (reviewed by Florini et al., 1996). Thyroid-stimulating hormone (TSH) induces IGF-I mRNA expression in thyroid cells (Hofbauer et al., 1995). Finally, estrogen may play an important role in the local regulation of IGF-I in ovarian and uterine tissue (Murphy and Friesen, 1988).

The role of circulating "endocrine" IGF-I (primarily derived from the liver) versus the local "paracrine/autocrine" IGF-I has recently been reevaluated through the use of the tissue-specific gene-deletion (cre-lox/P) technology (Yakar et al., 1999). In this system, a construct including the IGF-I gene flanked by lox/P sequences was introduced into mice using homologous recombination. In another transgenic mouse line, the recombinase enzyme (cre) is expressed in the tissue of interest using a specific promoter-enhancer element, and the two mice lines are crossed. Using this approach, IGF-I gene expression was ablated specifically in the liver of mice (Yakar et al., 1999). The resulting mice exhibited complete abrogation of IGF-I gene expression from the liver with normal production in all nonhepatic tissues. Although total circulating IGF-I levels were reduced by 75% to 80% in the "liver knockout" mice, their growth and development was apparently normal. However, the total levels of circulating IGF-I levels were further reduced when these mice were crossed with acid-labile subunit (ALS) gene-deleted mice. In the circulation, ALS forms a complex with IGFs and IGFBP-3. The loss of ALS results in very low binding capacity and protection for circulating IGFs. This double knockout animal exhibited growth retardation, suggesting that circulating IGF-I contributes to growth, but that local production of IGF was also important (Yakar et al., 2002). Interestingly, these studies revealed that IGF-I has dual effects on bone development; circulating IGF-I played a major role in bone density, whereas both circulating and local IGF-I were involved in linear bone growth.

### IGF-II

Ablation of the IGF-II gene demonstrated that IGF-II plays a critical role in normal growth and development in mice. From embryonic day 11 onward, IGF-II knockout mice exhibited proportionate growth retardation. There were no further postnatal effects on growth in these mice, because the expression and circulating levels of IGF-II decrease dramatically after birth in rodents.

In contrast, the major effects of IGF-I gene ablation were observed during the postnatal period, including severe growth retardation, infertility, and other effects (Baker et al., 1993; Liu et al., 1993; Liu and Le Roith, 1999; Wang et al., 1999). Expression of IGF-II in cultured cells is regulated by various agents, including follicle-stimulating hormone (FSH), chorionic gonadotrophin and cyclic AMP (cAMP) in ovarian cells, adrenocorticotropic hormone (ACTH) and cAMP in fetal adrenal cells, glucocorticoids and thyroid hormone in hepatic cells, and glucose in a pancreatic beta-cell line. IGF-II is also increased in response to glucose in fetal hepatocytes, and plays an important autocrine/paracrine role in skeletal muscle myoblast differentiation in vitro (Stewart and Rotwein, 1996). In the circulation, IGF-II is a 67–amino acid, single-chain polypeptide. However, patients with certain types of tumors occasionally release "big-IGF-II," a larger precursor form with a 21–amino acid extension designated as the E-peptide. Big-IGF-II may cause hypoglycemia by interfering with the normal effect of the IGFBPs on neutralizing circulating IGFs, thereby enabling big-IGF-II to interact with IRs (Daughaday et al., 1988).

### RECEPTORS

### IR and IGF-IR

The IR and IGF-IR are products of separate genes that span >100 kilobases and contain over 20 exons. They belong to the membrane spanning family of tyrosine kinase receptors. These receptors are organized into functional domains. The mature receptor is expressed in an $\alpha 2\beta 2$ configuration, where two $\alpha$ chains are joined by disulfide bonds. The $\beta$ subunit lies entirely within the extracellular region and contains a cysteine-rich domain that forms the primary binding site for IGFs in the IGF-IR, whereas insulin apparently binds regions flanking the cysteine-rich domain of the IR. The $\beta$ subunit includes a 24-residue hydrophobic transmembrane domain, a short extracellular region, and a large cytoplasmic region that includes a tyrosine kinase domain. The tyrosine kinase region is highly conserved between the IGF-IR and the IR, sharing approximately 84% similarity at the amino acid level. The juxtamembrane region contains various motifs that bind to important intracellular substrates. The most divergent region between the two receptors is the cytoplasmic carboxyl-terminal domain (Ulrich et al., 1985, 1986).

As a consequence of this high level of homology, hybrid receptors, comprised of an insulin $\alpha\beta$ hemireceptor and an IGF-I $\alpha\beta$ hemireceptor, can form in tissues and cultured cells expressing both the IR and the IGF-IR (Federici et al., 1997a, 1997b). Such hybrid receptors may play a role in the divergent actions of insulin and IGF-I. The biological response elicited by these hybrid receptors can vary, depending on the specific isoforms of the IR that are involved. The IR exists as two isoforms generated by alternative splicing of the IR gene that either lacks (IR-A) or includes (IR-B) 12 amino acid residues encoded by exon 11 at the carboxyl terminus of the IR $\alpha$ subunit. Hybrids comprised of an IR-A hemireceptor and an IGF-IR hemireceptor bound IGF-I, IGF-II, and insulin and exhibited cell proliferation and migration, even in response to insulin, presumably via activation of the IGF-IR. In contrast, hybrids comprised of an IR-B hemireceptor and an IGF-IR hemireceptor were only responsive to IGF-I but not IGF-II or insulin (Pandini et al., 2002). The IR-A homoreceptor binds IGF-II with high affinity and may be important in the functional role of IGF-II in the fetus and in dedifferentiated (malignant) cells (Frasca et al., 1999; Sciacca et al., 1999).

### Receptor Functioning

Najjar and coworkers identified pp120, a plasma membrane glycoprotein, as a specific substrate for the IR but not the IGF-IR (Najjar et al., 1997; Soni et al., 2000). Phosphorylation of pp120 is required for its function in insulin endocytosis (Formisano et al., 1995), and also for its inhibitory effect on the mitogenic actions of insulin (Soni et al., 2000). Interestingly, when the carboxyl terminus of the IGF-IR is replaced by an equivalent region of the IR, the chimeric IGF-IR can then bind to and phosphorylate pp120, and the effect of IGF-I on cell growth is decreased (Soni et al., 2000). Mutation of $Tyr^{1316}$ in the IR, which is not conserved in the IGF-IR, abrogates the insulin-induced tyrosine phosphorylation of pp120 and its ability to suppress insulin-induced mitogenesis.

Like the IR, IGF-IRs are internalized following ligand binding and activation. Activation of the IGF-IR enhances the association of EHD1 with the IGF-IR and SNAP29. EHD1 belongs to a family of proteins that contain EH domains and are involved in forming protein complexes that promote clathrin-coated vesicles and are involved in endocytosis. Overexpression of EHD1 in NIH-3T3 fibroblasts inhibits IGF-I signaling and supports the hypothesis that endocytosis may be a mechanism whereby the IGF-IR signal is abrogated (Rotem-Yehudar et al., 2001). Internalization is also affected by G$\alpha$i and $\beta$-arrestin-1, which bind to the IGF-IR after activation, and also enhances the activation of mitogen-activated protein (MAP) kinase by the IGF-IR (Dalle et al., 2001; Lin et al., 1998).

### SIGNAL TRANSDUCTION VIA IR AND IGF-IR

### Common Signaling Pathways

In addition to the structural similarities of the IR and IGF-IR, many of the intracellular signaling events that result from ligand-induced receptor activation are remarkably similar (Cheatham and Kahn, 1995; Le Roith et al., 1995; White, 1994). The tyrosine kinase domains of the IR and IGF-IR catalyze the phosphorylation of specific substrates and are critical for IR- and IGF-IR–induced signaling (Kato et al., 1993). All conserved tyrosine residues that are phosphorylated in the IR in response to insulin are also phosphorylated in the IGF-IR in response to IGF-I. Thus, the IGF-IR and IR share many substrates, such as the members of the IR substrate (IRS) family (IRS-1 to IRS-4), Gab-1, and Shc (Fantin et al., 1998; Lavan and Lienhard, 1993; Patti et al., 1995; Pelicci et al., 1992; Winnay et al., 2000). IRS-1 is the best characterized of the IRS family members. IRS proteins and Shc contain an amino-terminal phosphotyrosine-binding (PTB) domain that enables them to bind to the juxtamembrane domain of the IR and IGF-IR, via phosphotyrosines in NPEY motifs. IRS-1 and Shc are competitive substrates that can interact with both the IR and the IGF-IR (Sasaoka et al., 1996). Upon stimulation with insulin or IGF-I, tyrosine-phosphorylated IRS and Shc proteins engage in the formation of signaling complexes via phosphotyrosine-containing binding motifs (YXXM) within Src homology 2 (SH2) domains found in molecules like GRB2 (growth factor receptor binding-2 protein) (Lowenstein et al., 1992; Skolnik et al., 1993), and the p85 regulatory subunit of phosphatidylinositol 3′ kinase (PI3K) (Backer et al., 1992). The phosphotyrosine residues on IRS-1 form docking sites for additional signaling molecules, including Syp (SHPTP2) (Xiao et al., 1994), Fyn (Sun et al., 1996), Nck (Lee et al., 1993), and Crk (Beitner-Johnson et al., 1996).

By binding to GRB2, IRS proteins couple GRB2 to the IR or IGF-IR. Shc also couples these receptors to GRB2, even more strongly than the IRS proteins. Once associated with Shc and/or IRS proteins, GRB2 forms a complex with the Son of Sevenless (SOS) p21Ras guanine nucleotide GDP/GTP exchange factor. This causes translocation of SOS to the plasma membrane and activation of the Ras/MAP kinase pathway and regulation of cell growth, differentiation, and proliferation in response to insulin and IGF-I (Blenis, 1993; Crews and Erikson, 1993).

Activation of the IR and IGF-IR and their intracellular components in response to ligand binding is transient and is controlled by several mechanisms, including phosphorylation, dephosphorylation, and/or degradation of certain components. Syp (SHPTP2, PTP-1D, or SHP-2) (Lamothe et al., 1996; Maile and Clemmons, 2002) and PTP1B (Ravichandran et al., 2001) are two candidate phosphotyrosine phosphatases that interact with both the IGF-IR and IR and dephosphorylate them in response to the ligand binding. SHIP-2 and PTEN (Butler et al., 2002) are lipid phosphatases that play significant roles in the regulation of PI3K signaling in response to both IGF-I and insulin. It has also been shown that phosphorylation of the serine or threonine residues of IRS-1 (Rui et al., 2001) or degradation of IRS-1 (Sun et al., 1999) can counterregulate the insulin or IGF-1 response.

One of the major effects of IGF-I is to promote cell survival. Several molecular mechanisms underlying the basis of

IGF-I–mediated cell survival have been described, involving PI3K/Akt, MAP kinase, and 14-3-3 proteins. All of these proteins are associated with increases in the phosphorylation state of the proapoptotic protein BAD (Bai et al., 1999). In neuronal cells, IGF-I induced phosphorylation of the forkhead transcription factor via a PI3K/Akt-dependent signaling pathway, thereby inhibiting apoptosis. Furthermore, IGF-I promotes transcription of the antiapoptotic bcl-2 gene, by promoting phosphorylation of the cAMP response element–binding protein (CREB) transcription factor via both the p38 stress-activated protein kinase and PI3K/Akt pathways (Pugazhenthi et al., 1999). The antiapoptotic effects of IGF-I are mediated through PI3K and are also important in preventing mannitol-induced apoptosis. Mannitol induces dephosphorylation and degradation of FAK, apparently in response to activation of capsases. IGF-I counteracts this effect, leading to increases in cell adhesion and cell survival (Kim and Feldman, 2002). The IGF-IR can promote cell spreading and cell contact with the extracellular matrix (ECM) by interacting with RACK1, a G$\beta$ homologue. This interaction delays progression of the cell cycle, but enhances FAK and paxillin phosphorylation, thereby altering integrin signaling (Hermanto et al., 2002). RACK1 may also modulate the antiapoptotic effects of the IGF-IR via Akt (Kiely et al., 2002).

### Receptor Cross-Talk

The insulin and IGF-I receptors do not function in isolation; their signals affect and are affected by other receptor signaling cascades. The IGF-IR activates heterotetrameric G proteins in certain cell types. It has been reported that the $\beta\gamma$ subunits of the Gi class of G proteins can mediate IGF-I–induced activation of MAP kinase (Luttrell et al., 1995). In addition, it has been demonstrated that the IGF-IR forms a complex with the G$\alpha$ subunit of Gi proteins (Dalle et al., 2001). Interestingly, chronic treatment with insulin can cause heterologous desensitization of the IGF-I–induced activation of MAP kinase by down-regulation of $\beta$-arrestin-1, a molecule that plays an important role in IGF-IR internalization and activation of MAP kinase (Dalle et al., 2002). Other examples of this type of receptor cross-talk include the estrogen receptor and the IGF-IR. Thus, in MCF-7 breast cancer cell lines and other cell lines, costimulation with estradiol and IGF-I can induce either additive or synergistic effects on downstream signaling pathways, such as PI3K and various cell cycle events (Dupont et al., 2000). The IGF-IR interacts with the cell-cell adhesion complex that includes E-cadherin, $\beta$-catenin, and p120 catenin. When IGF-IR expression is reduced in MCF-7 cells by the introduction of antisense mRNA, these cells exhibit a more malignant phenotype that is associated with a reduction in the cell-cell adhesion complex. This is throught to result from a p120 catenin-induced reduction in E-cadherin and activation of Rac and Cdc42 activity (Pennisi et al., 2002).

### IGFBPs

To date, six IGFBPs that have high affinity for the IGFs have been described (Jones and Clemmons, 1995). These proteins are characterized by their well-conserved amino- and carboxyl-terminal domains that contain several highly conserved cysteine residues. IGFBPs are found both in the circulation and at the local tissue level. In the circulation, IGFBPs act as "transport proteins" for the IGFs, but at the local level they act as modulators of IGF activity (Zapf, 1995). In the circulation, the major proportion of IGF is bound to a 150-kDa complex that includes IGFBP-3 and ALS, which protect the IGFs from proteases and prolong their circulating half-life (Rajaram et al., 1997). IGFBPs may also function as carrier proteins, because other IGFBPs may contribute to a 50-kDa circulating complex that facilitates the transfer of IGFs from the circulation to target cells.

At the target cell level, the IGFBPs have multiple roles; some IGFBPs modulate the effects of the IGFs and others act independently from the IGFs and the IGF-IR (Lalou et al., 1996). IGFBP-induced inhibition of IGF-I action occurs when IGF-BPs prevent the interaction of IGFs with the IGF-IR (Baxter, 2000; Jones and Clemmons, 1995). However, the binding affinities of IGFBPs are altered by various modifications, including phosphorylation, partial proteolysis, and attachment to the cell surface or ECM. For example, dephosphorylation of IGFBP-1 lowers its affinity for IGFs. Attachment of IGFBP-3 to the cell surface or IGFBP-5 to the ECM lowers their respective affinities for IGFs. All of these effects have been proposed to enhance the delivery of IGFs to the IGF-IR.

The potential role of the IGFBP system that has been most extensively studied as it relates to complications of diabetes is its effects on nephropathy. GH and IGF-I were initially shown to affect the diabetic kidney by increasing glomerular filtration rates and renal plasma flow, resulting in the typical enlarged kidney seen in patients with recent onset of diabetes (Flyvbjerg, 2000). Similar changes were seen in experimental animal models of diabetes, particularly of the type 1 variety. This change was associated with increased IGF-I concentrations in the renal tissue, despite a significant reduction in IGF-I gene expression as measured by mRNA levels. This suggested that the IGF-I peptide was being trapped from the circulation. Indeed, subsequent studies demonstrated that both the IGF-IR and certain IGFBPs were expressed by the kidney at higher levels than in control animals and suggested that the IGFBPs play a critical role in the presentation of IGF-I to its receptor and thereby enhance its biological function (Landau et al., 1995; Werner et al., 1990). Interestingly, somatstatin analoges have been shown to inhibit IGFBP gene expression in diabetic animals and to prevent the early renal changes described above (Raz et al., 1998).

IGFBP-1 has been reported to exhibit IGF-independent actions. When the RGD sequence was prevented from interacting with the integrin $\alpha5\beta1$ receptor, IGFBP-1 was unable to stimulate cell migration (Jones et al., 1993). In breast cancer cells, the binding of IGFBP-1 to integrin at the cell surface resulted in dephosphorylation of FAK, detachment from the ECM, and cellular apoptosis (Perks et al., 1999). A proapoptotic action of IGFBP-3 has also been reported to be independent of IGF (Perks et al., 1999a, 1999b; Butt et al., 2000; Maile et al., 1999). A number of potential IGF-independent survival-promoting effects of IGFBP-4 and IGFBP-5 have been reported; however, the mechanisms underlying these effects are not known (Perks et al., 1999a). Finally, IGFBP-3 and IGFBP-5 are translocated into the nucleus via the importin-5 subunit (Schedlich et al., 1998, 2000). The cellular consequences of these actions are unknown. Nevertheless, taken together, these findings suggest that various IGF-I–independent actions of IGFBPs can regulate cell growth and survival.

### SUMMARY

The IGF system is ubiquitous and has multiple roles in normal physiology and pathological states. Although it regulates important functions in normal growth, development, and differentiation of most tissues, aberrations in the IGF system are clearly associated with various pathological conditions, including cancer, acromegaly, growth retardation, diabetes, and its associated complications, such as retinopathy, nephropathy, neuropathy, and insulin resistance. Understanding the control of expression of the various components as well as the signal transduction pathways involved in IGF-I receptor function will facilitate the development of specific therapeutic modalities for these and other disorders.

### REFERENCES

Backer, J. M., Myers, M. G., Jr., Shoelson, S. E., Chin, D. J., Sun, X. J., Miralpeix, M., Hu, P., Margolis, B., Skolnik, E. Y., Schlessinger, J., et al. (1992) Phosphatidylinositol 3′-kinase is activated by association with IRS-1 during insulin stimulation. *EMBO J,* **11,** 3469–3479.

Bai, H., Pollman, M. J., Inishi, Y., and Gibbons, G. H. (1999) Regulation of vascular smooth muscle cell apoptosis. Modulation of bad by a phosphatidylinositol 3-kinase-dependent pathway. *Circ. Res.,* **85,** 229–237.

Baker, J., Liu, J. P., Robertson, E. J., and Efstratiadis, A. (1993) Role of insulin-like growth factors in embryonic and postnatal growth. *Cell,* **75,** 73–82.

Baxter, R. C. (2000) The IGF binding proteins. *Am. J. Physiol. Endocrinol. Metab.,* **278,** E967–E976.

Beitner-Johnson, D., Blakesley, V. A., Shen-Orr, Z., Jimenez, M., Stannard, B., Wang, L. M., Pierce, J., and Le Roith, D. (1996) The protooncogene product c-Crk associates with insulin receptor substrate-1 and 4PS. Modulation by insulin growth factor-I (IGF) and enhanced IGF-I signaling. *J. Biol. Chem.,* **271,** 9287–9290.

Bichell, D. P., Kikuchi, K., and Rotwein, P. (1992) Growth hormone rapidly activates insulin-like growth factor I gene transcription in vivo. *Mol. Endocrinol.,* **6,** 1899–1908.

Blenis, J. (1993) Signal transduction via the MAP kinases: Proceed at your own RSK. *Proc. Natl. Acad. Sci. U. S. A.,* **90,** 5889–5892.

Brink, M., Chrast, J., Price, S. R., Mitch, W. E., and Delafontaine, P. (1999) Angiotensin II stimulates gene expression of cardiac insulin-like growth factor I and its receptor through effects on blood pressure and food intake. *Hypertension,* **34,** 1053–1059.

Butler, M., McKay, R. A., Popoff, I. J., Gaarde, W. A., Witchell, D., Murray, S. F., Dean, N. M., Bhanot, S. and Monia, B. P. (2002) Specific inhibition of PTEN expression reverses hyperglycemia in diabetic mice. *Diabetes,* **51,** 1028–1034.

Butt, A. J., Firth, S. M., King, M. A., and Baxter, R. C. (2000) Insulin-like growth factor-binding protein-3 modulates expression of Bax and Bcl-2 and potentiates p53-independent radiation-induced apoptosis in human breast cancer cells. *J. Biol. Chem.,* **275,** 39174–39181.

Cheatham, B., and Kahn, C. R. (1995) Insulin action and the insulin signaling network. *Endocr. Rev.,* **16,** 117–142.

Clemmons, D. R. (2001) Use of mutagenesis to probe IGF-binding protein structure/function relationships. *Endocr. Rev.,* **22,** 800–817.

Crews, C. M., and Erikson, R. L. (1993) Extracellular signals and reversible protein phosphorylation: What to Mek of it all. *Cell,* **74,** 215–217.

Dalle, S., Imamura, T., Rose, D. W., Worrall, D. S., Ugi, S., Hupfeld, C. J., and Olefsky, J. M. (2002) Insulin induces heterologous desensitization of G-protein-coupled receptor and insulin-like growth factor I signaling by downregulating beta-arrestin-1. *Mol. Cell Biol.,* **22,** 6272–6285.

Dalle, S., Ricketts, W., Imamura, T., Vollenweider, P., and Olefsky, J. M. (2001) Insulin and insulin-like growth factor I receptors utilize different G protein signaling components. *J. Biol. Chem.,* **276,** 15688–15695.

Daughaday, W. H., Emanuele, M. A., Brooks, M. H., Barbato, A. L., Kapadia, M., and Rotwein, P. (1988) Synthesis and secretion of insulin-like growth factor II by a leiomyosarcoma with associated hypoglycemia. *N. Engl. J. Med.,* **319,** 1434–1440.

Dupont, J., Karas, M., and Le Roith, D. (2000) The potentiation of estrogen on insulin-like growth factor I action in MCF-7 human breast cancer cells includes cell cycle components. *J. Biol. Chem.,* **275,** 35893–35901.

Ernst, M., and Rodan, G. A. (1991) Estradiol regulation of insulin-like growth factor-I expression in osteoblastic cells: Evidence for transcriptional control. *Mol. Endocrinol.,* **5,** 1081–1089.

Fantin, V. R., Sparling, J. D., Slot, J. W., Keller, S. R., Lienhard, G. E., and Lavan, B. E. (1998) Characterization of insulin receptor substrate 4 in human embryonic kidney 293 cells. *J. Biol. Chem.,* **273,** 10726–10732.

Federici, M., Porzio, O., Zucaro, L., Fusco, A., Borboni, P., Lauro, D., and Sesti, G. (1997a) Distribution of insulin/insulin-like growth factor-I hybrid receptors in human tissues. *Mol. Cell Endocrinol.,* **129,** 121–126.

Federici, M., Porzio, O., Zucaro, L., Giovannone, B., Borboni, P., Marini, M. A., Lauro, D., and Sesti, G. (1997b) Increased abundance of insulin/IGF-I hybrid receptors in adipose tissue from NIDDM patients. *Mol. Cell Endocrinol.,* **135,** 41–47.

Florini, J. R., Ewton, D. Z., and Coolican, S. A. (1996) Growth hormone and the insulin-like growth factor system in myogenesis. *Endocr. Rev.,* **17,** 481–517.

Flyvbjerg, A. (2000) Putative pathophysiological role of growth factors and cytokines in experimental diabetic kidney disease. *Diabetologia,* **43,** 1205–1223.

Formisano, P., Najjar, S. M., Gross, C. N., Philippe, N., Oriente, F., Kern-Buell, C. L., Accili, D., and Gorden, P. (1995) Receptor-mediated internalization of insulin. Potential role of pp120/HA4, a substrate of the insulin receptor kinase. *J. Biol. Chem.,* **270,** 24073–24077.

Frasca, F., Pandini, G., Scalia, P., Sciacca, L., Mineo, R., Costantino, A., Goldfine, I. D., Belfiore, A., and Vigneri, R. (1999) Insulin receptor isoform A, a newly recognized, high-affinity insulin-like growth factor II receptor in fetal and cancer cells. *Mol. Cell Biol.,* **19,** 3278–3288.

Hermanto, U., Zong, C. S., Li, W., and Wang, L. H. (2002) RACK1, an insulin-like growth factor I (IGF-I) receptor-interacting protein, modulates IGF-I-dependent integrin signaling and promotes cell spreading and contact with extracellular matrix. *Mol. Cell Biol.,* **22,** 2345–2365.

Hofbauer, L. C., Rafferzeder, M., Janssen, O. E., and Gartner, R. (1995) Insulin-like growth factor I messenger ribonucleic acid expression in porcine thyroid follicles is regulated by thyrotropin and iodine. *Eur. J. Endocrinol.,* **132,** 605–610.

Jones, J. I., and Clemmons, D. R. (1995) Insulin-like growth factors and their binding proteins: Biological actions. *Endocr. Rev.,* **16,** 3–34.

Jones, J. I., Gockerman, A., Busby, W. H., Jr., Wright, G., and Clemmons, D. R. (1993) Insulin-like growth factor binding protein 1 stimulates cell migration and binds to the alpha 5 beta 1 integrin by means of its Arg-Gly-Asp sequence. *Proc. Natl. Acad. Sci. U. S. A.,* **90,** 10553–10557.

Kato, H., Faria, T. N., Stannard, B., Roberts, C. T., Jr., and Le Roith, D. (1993) Role of tyrosine kinase activity in signal transduction by the insulin-like growth factor-I (IGF-I) receptor. Characterization of kinase-deficient IGF-I receptors and the action of an IGF-I-mimetic antibody (alpha IR-3). *J. Biol. Chem.,* **268,** 2655–2661.

Kaytor, E. N., Zhu, J. L., Pao, C. I., and Phillips, L. S. (2001) Insulin-responsive nuclear proteins facilitate Sp1 interactions with the insulin-like growth factor-I gene. *J. Biol. Chem.,* **276,** 36896–36901.

Kiely, P. A., Sant, A., and O'Connor, R. (2002) RACK1 is an insulin-like growth factor 1 (IGF-1) receptor-interacting protein that can regulate IGF-1-mediated Akt activation and protection from cell death. *J. Biol. Chem.,* **277,** 22581–22589.

Kim, B., and Feldman, E. L. (2002) Insulin-like growth factor I prevents mannitol-induced degradation of focal adhesion kinase and Akt. *J. Biol. Chem.,* **277,** 27393–27400.

Lalou, C., Lassarre, C., and Binoux, M. (1996) A proteolytic fragment of insulin-like growth factor (IGF) binding protein-3 that fails to bind IGFs inhibits the mitogenic effects of IGF-I and insulin. *Endocrinology,* **137,** 3206–3212.

Lamothe, B., Bucchini, D., Jami, J., and Joshi, R. L. (1996) Reexamining interaction of the SH2 domains of SYP and GAP with insulin and IGF-1 receptors in the two-hybrid system. *Gene,* **182,** 77–80.

Landau, D., Chin, E., Bondy, C., Domene, H., Roberts, C. T., Jr., Gronbaek, H., Flyvbjerg, A., and Le Roith, D. (1995) Expression of insulin-like growth factor binding proteins in the rat kidney: Effects of long-term diabetes. *Endocrinology,* **136,** 1835–1842.

Lavan, B. E., and Lienhard, G. E. (1993) The insulin-elicited 60-kDa phosphotyrosine protein in rat adipocytes is associated with phosphatidylinositol 3-kinase. *J. Biol. Chem.,* **268,** 5921–5928.

Lee, C. H., Li, W., Nishimura, R., Zhou, M., Batzer, A. G., Myers, M. G., Jr., White, M. F., Schlessinger, J., and Skolnik, E. Y. (1993) Nck associates with the SH2 domain-docking protein IRS-1 in insulin-stimulated cells. *Proc. Natl. Acad. Sci. U. S. A.,* **90,** 11713–11717.

Le Roith, D., Bondy, C., Yakar, S., Liu, J. L., and Butler, A. (2001) The somatomedin hypothesis: 2001. *Endocr. Rev.,* **22,** 53–74.

Le Roith, D., Werner, H., Beitner-Johnson, D., and Roberts, C. T., Jr. (1995) Molecular and cellular aspects of the insulin-like growth factor I receptor. *Endocrine Rev.,* **16,** 143–163.

Lin, F. T., Daaka, Y., and Lefkowitz, R. J. (1998) Beta-arrestins regulate mitogenic signaling and clathrin-mediated endocytosis of the insulin-like growth factor I receptor. *J. Biol. Chem.,* **273,** 31640–31643.

Liu, J. L., and Le Roith, D. (1999) Insulin-like growth factor I is essential for postnatal growth in response to growth hormone. *Endocrinology,* **140,** 5178–5184.

Liu, J. P., Baker, J., Perkins, A. S., Robertson, E. J., and Efstratiadis, A. (1993) Mice carrying null mutations of the genes encoding insulin-like growth factor I (Igf-1) and type 1 IGF receptor (Igf1r). *Cell,* **75,** 59–72.

Lowenstein, E. J., Daly, R. J., Batzer, A. G., Li, W., Margolis, B., Lammers, R., Ullrich, A., Skolnik, E. Y., Bar-Sagi, D., and Schlessinger, J. (1992) The SH2 and SH3 domain-containing protein GRB2 links receptor tyrosine kinases to ras signaling. *Cell,* **70,** 431–442.

Luttrell, L. M., van Biesen, T., Hawes, B. E., Koch, W. J., Touhara, K., and Lefkowitz, R. J. (1995) G beta gamma subunits mediate mitogen-activated protein kinase activation by the tyrosine kinase insulin-like growth factor 1 receptor. *J. Biol. Chem.,* **270,** 16495–16498.

Maile, L. A., and Clemmons, D. R. (2002) Regulation of insulin-like growth factor I receptor dephosphorylation by SHPS-1 and the tyrosine phosphatase SHP-2. *J. Biol. Chem.,* **277,** 8955–8960.

Maile, L. A., Gill, Z. P., Perks, C. M., and Holly, J. M. (1999) The role of cell surface attachment and proteolysis in the insulin-like growth factor (IGF)-independent effects of IGF-binding protein-3 on apoptosis in breast epithelial cells. *Endocrinology,* **140,** 4040–4045.

Mulroney, S. E., Lumpkin, M. D., Roberts, C. T., Jr., Le Roith, D., and Haramati, A. (1992) Effect of a growth hormone-releasing factor antagonist on compensatory renal growth, insulin-like growth factor-I (IGF-I), and IGF-I receptor gene expression after unilateral nephrectomy in immature rats. *Endocrinology,* **130,** 2697–2702.

Murphy, L. J., and Friesen, H. G. (1988) Differential effects of estrogen and growth hormone on uterine and hepatic insulin-like growth factor I gene expression in the ovariectomized hypophysectomized rat. *Endocrinology,* **122,** 325–332.

Najjar, S. M., Blakesley, V. A., Li Calzi, S., Kato, H., Le Roith, D., and Choice, C. V. (1997) Differential phosphorylation of pp120

by insulin and insulin-like growth factor-1 receptors: Role for the C-terminal domain of the beta-subunit. *Biochemistry,* **36,** 6827–6834.

Pandini, G., Frasca, F., Mineo, R., Sciacca, L., Vigneri, R., and Belfiore, A. (2002) Insulin/insulin-like growth factor I hybrid receptors have different biological characteristics depending on the insulin receptor isoform involved. *J. Biol. Chem.,* **277,** 39684–39695.

Patti, M. E., Sun, X. J., Bruening, J. C., Araki, E., Lipes, M. A., White, M. F., and Kahn, C. R. (1995) 4PS/insulin receptor substrate (IRS)-2 is the alternative substrate of the insulin receptor in IRS-1-deficient mice. *J. Biol. Chem.,* **270,** 24670–24673.

Pelicci, G., Lanfrancone, L., Grignani, F., McGlade, J., Cavallo, F., Forni, G., Nicoletti, I., Pawson, T., and Pelicci, P. G. (1992) A novel transforming protein (SHC) with an SH2 domain is implicated in mitogenic signal transduction. *Cell,* **70,** 93–104.

Pennisi, P. A., Barr, V., Nunez, N. P., Stannard, B., and Le Roith, D. (2002) Reduced expression of insulin-like growth factor I receptors in MCF-7 breast cancer cells leads to a more metastatic phenotype. *Cancer Res.,* **62,** 6529–6537.

Perks, C. M., Bowen, S., Gill, Z. P., Newcomb, P. V., and Holly, J. M. (1999a) Differential IGF-independent effects of insulin-like growth factor binding proteins (1–6) on apoptosis of breast epithelial cells. *J. Cell Biochem.,* **75,** 652–664.

Perks, C. M., Newcomb, P. V., Norman, M. R., and Holly, J. M. (1999b) Effect of insulin-like growth factor binding protein-1 on integrin signaling and the induction of apoptosis in human breast cancer cells. *J. Mol. Endocrinol.,* **22,** 141–150.

Pugazhenthi, S., Boras, T., O'Connor, D., Meintzer, M. K., Heidenreich, K. A., and Reusch, J. E. (1999) Insulin-like growth factor I-mediated activation of the transcription factor cAMP response element-binding protein in PC12 cells. Involvement of p38 mitogen-activated protein kinase-mediated pathway. *J. Biol. Chem.,* **274,** 2829–2837.

Rajaram, S., Baylink, D. J., and Mohan, S. (1997) Insulin-like growth factor-binding proteins in serum and other biological fluids: Regulation and functions. *Endocr. Rev.,* **18,** 801–831.

Ravichandran, L. V., Chen, H., Li, Y., and Quon, M. J. (2001) Phosphorylation of PTP1B at Ser(50) by Akt impairs its ability to dephosphorylate the insulin receptor. *Mol. Endocrinol.,* **15,** 1768–1780.

Raz, I., Rubinger, D., Popovtzer, M., Gronbaek, H., Weiss, O., and Flyvbjerg, A. (1998) Octreotide prevents the early increase in renal insulin-like growth factor binding protein 1 in streptozotocin diabetic rats. *Diabetes,* **47,** 924–930.

Rotem-Yehudar, R., Galperin, E., and Horowitz, M. (2001) Association of insulin-like growth factor 1 receptor with ehd1 and snap29. *J. Biol. Chem.,* **276,** 33054–33060.

Rui, L., Aguirre, V., Kim, J. K., Shulman, G. I., Lee, A., Corbould, A., Dunaif, A., and White, M. F. (2001) Insulin/IGF-1 and TNF-alpha stimulate phosphorylation of IRS-1 at inhibitory Ser307 via distinct pathways. *J. Clin. Invest.,* **107,** 181–189.

Sasaoka, T., Ishiki, M., Sawa, T., Ishihara, H., Takata, Y., Imamura, T., Usui, I., Olefsky, J. M., and Kobayashi, M. (1996) Comparison of the insulin and insulin-like growth factor 1 mitogenic intracellular signaling pathways. *Endocrinology,* **137,** 4427–4434.

Schedlich, L. J., Le Page, S. L., Firth, S. M., Briggs, L. J., Jans, D. A., and Baxter, R. C. (2000) Nuclear import of insulin-like growth factor-binding protein-3 and -5 is mediated by the importin beta subunit. *J. Biol. Chem.,* **275,** 23462–23470.

Schedlich, L. J., Young, T. F., Firth, S. M., and Baxter, R. C. (1998) Insulin-like growth factor-binding protein (IGFBP)-3 and IGFBP-5 share a common nuclear transport pathway in T47D human breast carcinoma cells. *J. Biol. Chem.,* **273,** 18347–18352.

Sciacca, L., Costantino, A., Pandini, G., Mineo, R., Frasca, F., Scalia, P., Sbraccia, P., Goldfine, I. D., Vigneri, R., and Belfiore, A. (1999) Insulin receptor activation by IGF-II in breast cancers: Evidence for a new autocrine/paracrine mechanism. *Oncogene,* **18,** 2471–2479.

Skolnik, E. Y., Lee, C. H., Batzer, A., Vicentini, L. M., Zhou, M., Daly, R., Myers, M. J., Jr., Backer, J. M., Ullrich, A., White, M. F., et al. (1993) The SH2/SH3 domain-containing protein GRB2 interacts with tyrosine-phosphorylated IRS1 and Shc: Implications for insulin control of ras signalling. *EMBO J.,* **12,** 1929–1936.

Soni, P., Lakkis, M., Poy, M. N., Fernstrom, M. A., and Najjar, S. M. (2000) The differential effects of pp120 (Ceacam 1) on the mitogenic action of insulin and insulin-like growth factor 1 are regulated by the nonconserved tyrosine 1316 in the insulin receptor. *Mol. Cell Biol.,* **20,** 3896–3905.

Stewart, C. E., and Rotwein, P. (1996) Growth, differentiation, and survival: Multiple physiological functions for insulin-like growth factors. *Physiol. Rev.,* **76,** 1005–1026.

Sun, X. J., Goldberg, J. L., Qiao, L. Y., and Mitchell, J. J. (1999) Insulin-induced insulin receptor substrate-1 degradation is mediated by the proteasome degradation pathway. *Diabetes,* **48,** 1359–1364.

Sun, X. J., Pons, S., Asano, T., Myers, M. G., Jr., Glasheen, E., and White, M. F. (1996) The Fyn tyrosine kinase binds Irs-1 and forms a distinct signaling complex during insulin stimulation. *J. Biol. Chem.,* **271,** 10583–10587.

Ullrich, A., Bell, J. R., Chen, E. Y., Herrera, R., Petruzzelli, L. M., Dull, T. J., Gray, A., Coussens, L., Liao, Y. C., Tsubokawa, M., et al. (1985) Human insulin receptor and its relationship to the tyrosine kinase family of oncogenes. *Nature,* **313,** 756–761.

Ullrich, A., Gray, A., Tam, A. W., Yang-Feng, T., Tsubokawa, M., Collins, C., Henzel, W., Le Bon, T., Kathuria, S., Chen, E., et al. (1986) Insulin-like growth factor I receptor primary structure: Comparison with insulin receptor suggests structural determinants that define functional specificity. *EMBO J.,* **5,** 2503–2512.

Wang, J., Zhou, J., Powell-Braxton, L., and Bondy, C. (1999) Effects of Igf1 gene delection on postnatal growth patterns. *Endocrinology,* **140,** 3391–3394.

Werner, H., Shen-orr, Z., Stannard, B., Burguera, B., Roberts, C. T., Jr., and Le Roith, D. (1990) Experimental diabetes increases insulinlike growth factor I and II receptor concentration and gene expression in kidney. *Diabetes,* **39,** 1490–1497.

White, M. F. (1994) The IRS-1 signaling system. *Curr. Opin. Genet. Dev.,* **4,** 47–54.

Winnay, J. N., Bruning, J. C., Burks, D. J., and Kahn, C. R. (2000) Gab-1-mediated IGF-1 signaling in IRS-1-deficient 3T3 fibroblasts. *J. Biol. Chem.,* **275,** 10545–10550.

Xiao, S., Rose, D. W., Sasaoka, T., Maegawa, H., Burke, T. R., Jr., Roller, P. P., Shoelson, S. E., and Olefsky, J. M. (1994) Syp (SH-PTP2) is a positive mediator of growth factor-stimulated mitogenic signal transduction. *J. Biol. Chem.,* **269,** 21244–21248.

Yakar, S., Liu, J. L., Stannard, B., Butler, A., Accili, D., Sauer, B., and Le Roith, D. (1999) Normal growth and development in the absence of hepatic insulin-like growth factor I. *Proc. Natl. Acad. Sci. U. S. A.,* **96,** 7324–7329.

Yakar, S., Rosen, C. J., Beamer, W. G., Ackert-Bicknell, C. L., Wu, Y., Liu, J. L., Ooi, G. T., Setser, J., Frystyk, J., Boisclair, Y. R., and Le Roith, D. (2002) Circulating levels of IGF-1 directly regulate bone growth and density. *J. Clin. Invest.,* **110,** 771–781.

Zhang, J., Chrysis, D., and Underwood, L. E. (1998) Reduction of hepatic insulin-like growth factor I (IGF-I) messenger ribonucleic acid (mRNA) during fasting is associated with diminished splicing of IGF-I pre-mRNA and decreased stability of cytoplasmic IGF-I mRNA. *Endocrinology,* **139,** 4523–4530.

Zapf, J. (1995) Physiological role of the insulin-like growth factor binding proteins. *Eur. J. Endocrinol.,* **132,** 645–654.

